# The Classification of Movement in Infants for the Autonomous Monitoring of Neurological Development

**DOI:** 10.3390/s23104800

**Published:** 2023-05-16

**Authors:** Alexander Turner, Stephen Hayes, Don Sharkey

**Affiliations:** 1 Department of Computer Science, University of Nottingham, Nottingham NG8 1BB, UK; 2 Department of Engineering, Nottingham Trent University, Nottingham NG4 2EA, UK; steve.hayes@ntu.ac.uk; 3 Department of Medicine, University of Nottingham, Nottingham NG7 2RD, UK; don.sharkey@nottingham.ac.uk

**Keywords:** neurological development, infant development, deep learning, autonomous monitoring, movement assessment of infants

## Abstract

Neurodevelopmental delay following extremely preterm birth or birth asphyxia is common but diagnosis is often delayed as early milder signs are not recognised by parents or clinicians. Early interventions have been shown to improve outcomes. Automation of diagnosis and monitoring of neurological disorders using non-invasive, cost effective methods within a patient’s home could improve accessibility to testing. Furthermore, said testing could be conducted over a longer period, enabling greater confidence in diagnoses, due to increased data availability. This work proposes a new method to assess the movements in children. Twelve parent and infant participants were recruited (children aged between 3 and 12 months). Approximately 25 min 2D video recordings of the infants organically playing with toys were captured. A combination of deep learning and 2D pose estimation algorithms were used to classify the movements in relation to the children’s dexterity and position when interacting with a toy. The results demonstrate the possibility of capturing and classifying children’s complexity of movements when interacting with toys as well as their posture. Such classifications and the movement features could assist practitioners to accurately diagnose impaired or delayed movement development in a timely fashion as well as facilitating treatment monitoring.

## 1. Introduction

Neurological development in infants is a complex process with many factors influencing the typical development of the infant. Neurological disorders are defined as disorders that affect the brain, nerves and spinal cord, many of which are visible in early life [1,2,3]. Such disorders are often life long, have a significant impact on a person’s life experiences and require continued healthcare/treatment. Early diagnosis and intervention facilitate understanding for parents and improved opportunities for development in patients [4,5,6]. Unfortunately, movement disorders and neurological developmental delays can manifest in reduced capacity for activities of daily living, challenges with mobility and negative or reduced interactions with other people. It is possible that early diagnosis and intervention may enable the exploitation of the higher levels of neuroplasticity present in infants to enhance therapeutic interventions [7,8]. Numerous neurological disorders present in infants, these include but are not limited to, ataxia [9], autism [10], Functional Neurological Disorder [11] and Cerebral Palsy [7,8].

Functional Neurological Disorder and autism are both disorders that affect brain development and can have life-long consequences for patients [9,10]. Cerebral palsy (CP) is a life-long neurological condition that typically affects movement, coordination, balance and walking; other symptoms such as speech, hearing and vision problems can also occur [7,8,12]. CP is caused by damage to the infant’s brain prior to, during or soon after birth as a result of infection, stroke, a head injury or oxygen deprivation to the brain [12]. Approximately 2.1 per 1000 live births [13] are affected by CP. The risk of CP increases with preterm birth. Infants born between 32–36 weeks present with a rate of 4.4 per 1000 births. Those born between 28–31 weeks present with a rate of 38 per 1000 births and those born at less than 28 weeks have a rate of 86.4 per 1000 births [14]. CP is most commonly diagnosed when there is an absence of typical development detected, such as late crawling and walking. Signs of this, although common trends are often difficult to spot which means that the average infant receives a diagnosis around 19 months of age [5]. The difficulty of diagnosing CP and many other neurological disorders in infants is that diagnosis is often based on an absence rather than a positive indicator. Essentially, a delay in typical movement development, i.e., not being able to sit up independently, or a delay in crawling are common indicators [12]. As infants all develop at their own individual rate, early indicators are often very challenging to identify especially with the limited time heath care professionals are able to spend with the infant. With CP, as with other neurological disorders, it has been shown that early interventions which look to reduce the symptoms of CP by optimising the neurodevelopment of the infant are positive [7,8].

To try and reduce the impact of missed or late diagnosis, there has been a significant effort in recent decades to automate the diagnosis and monitoring of disease. The aim is to improve diagnostic accuracy and speed, providing healthcare professionals with better information, leading to improved decision making and ultimately, a reduced burden on healthcare services. Machine learning architectures, specifically, deep neural networks are well suited to classification of both visual and time series data [15,16,17,18]. Since their inception, deep learning architectures have outperformed humans in a number of medical classification tasks [19]. Furthermore, neural networks offer the potential for an “always on” diagnostic tool which does not require a face to face interaction between patients and practitioners, offering remote diagnostic and monitoring potential, as long as data are available. As a result of this work, at-home assessments are possible with trained medical professionals only needing to confirm rather than interpret results [16,19,20,21,22,23,24].

Pose estimation is the ability to quantify a pose in a given environment (Table 1). There has been significant work on this topic in recent decades, as there are many different fields in which such technology can be positive [25,26,27,28]. In recent years, methods have become more accurate and more efficient and therefore are available to run on a wider range of hardware such as smart phones [27,29]. For real-time monitoring, mediapipe [27] by Google (Table 1) is able to run at 24 frames per second on mobile phones and smaller computer systems such as the jetson-nano. Mediapipe has also been shown to be very effective [27], and would likely be effective in the pose estimation of children.

Deep learning has been successfully used to diagnose or monitor children’s movement patterns with respect to neurological disorders [20,21,30,31]. The primary aim of this paper is to demonstrate the feasibility of the in-home monitoring of children’s neurological development through 2D video recordings, 2D pose estimation and deep learning architectures [23,32,33]. Secondary aims are to assess the capacity of different deep learning architectures to optimally classify the time series movement data provided. Media pipe [27] was chosen for the 2D pose estimation as it is capable of running on devices with limited computational power such as mobile phones and is capable of generating time series data that can be used by the deep learning architectures.

There has been a significant effort to automate the process of diagnosing movement disorders using machine learning [22,34] and in recent years this has encompassed a push towards automating, diagnosing and monitoring infants with movement disorders. Some methods in this work focus on Deoxyribonucleic acid (DNA) sequencing or magnetic resonance imaging (MRI) scans to better achieve these outcomes [35,36]. However, most pertinent to this work are those studies which use visual methods in combination with deep learning to better understand the movements of children. There are multiple works that look specifically at automating the Prechtl General Movements Assessment (GMA) [20,37,38]. The GMA has been successful at classifying CP with a 98% sensitivity and a 91% specificity rate. However, there are complications associated with the differentiation of writhing and fidgety movements, which can lead to false positives and therefore cannot be considered in isolation [39]. The GMA involves the measurement of specific movements associated with the development of CP such as the tonic neck reflex, grasp reflex and rooting reflex. These works [20,37,38] use 2D pose estimation techniques in combination with deep learning to better understand the joint movements and positions over time to provide a GMA alternative. They use a combination of two data sets. The first being the MINI-RGBD data set, which is publicly available and consists of 1000 frame samples of infants on their backs moving over time. The second data set that is the RVI-25 dataset which was recorded as part of routine clinical care at the NHS Royal Victoria Infirmary, Newcastle upon Tyne, UK [38]. These data sets consist of recordings when the infants are at rest and not interacting with any objects or toys, which in terms of environmental settings is how the GMA test is conducted.

A different data set was used in the work by Groos, et al. [37] consisting of data from 557 infants with a high risk of perinatal brain injury. The identification of spontaneous, fidgety and writhing characteristics and movements were used as a predictor for the GMA. More generally, these articles look at limb positions and joint angels over time to deduce an estimate of the GMA score. A central characteristic of these works is the use of deep learning, and specifically recurrent neural networks or convolutional neural networks, or combinations of the two. Silva, et al. [40], undertook a scoping review of the automation of the GMA using video-based approaches. This revealed that the majority of machine learning techniques used when automating the GMA focus on statistical methods such as support vector machines, logistic regressions and k-means. Of the 40 studies identified, those using deep learning methods were outnumbered by the use of statistical methods 35 to 5.

The work on automating the GMA has been very successful, as has the human-conducted GMA. However, the GMA has a specific role and that is to assess the risk of CP in infants up to 20 weeks of age. The GMA does not consider a child’s interactions with their environment or with toys, a time at which children are most likely to maximise movement. Furthermore, the GMA is focused on diagnosis and not on monitoring development.

The work proposed in this article differs from other works and is novel for the following reasons:

- A novel data set has been created where infants between 3 and 12 months old were recorded organically both at rest and when interacting with toys. Therefore, we can see a wider range of hand movements alongside general positional and postural orientations such as laying down and sitting.
- Multiple deep learning architectures, including LSTM’s, Bi-LSTM’s and convolutional neural networks (CNN), as well as the Bi-LSTM-CNN have been used to ascertain which model optimally fits the data.
- The data were labelled in a human-readable way. Therefore, the frequency of specific movements or positions could be calculated. This, for example, allowed for output showing how often an infant used both hands to interact with a toy and whether or not they were capable of independently sitting upright or changing their position.
- The work demonstrates that Google’s media pipe is capable of accurately tracking the dexterous and positional movements of children when interacting with toys, and that this data can be classified using deep learning architectures.

## 2. Materials and Methods

**Figure 1:**
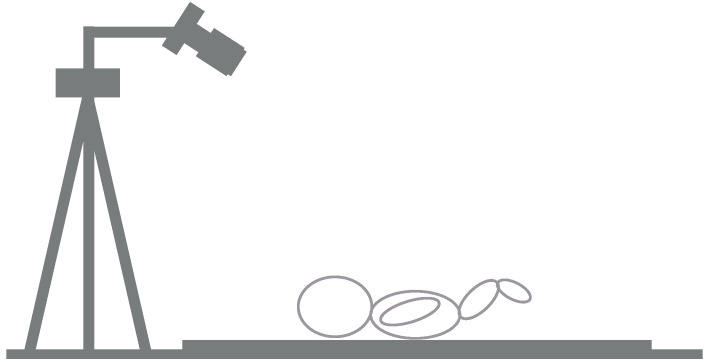
An illustration of how the camera is positioned relative to the child.

Twelve participants of mixed genders with no known neurological conditions aged between 3–12 months were placed upon a play mat and recorded when playing organically with toys. Ethical approval was gained from the department of computer science at Nottingham University (Approval number: CS-2020-R-73). The planned experimental time was a maximum of one hour per participant, with typical movement recordings lasting 16–43 min, depending on the infant’s emotional state and time-constrains of the parents. The infant is placed on the baby mat, but is free to move around as desired (Figure 1). Because of this, a top down view was best to ensure functionality of the 2D post estimation algorithm. The infant was recorded whilst playing with a range of toys aimed to encourage both dexterous and general movement. A Cannon EOS 70 digital video camera was used, at 25 fps with a wide angle lens (640 × 480 resolution).

The data labelling was split into two categories, the general positioning of the infant and dexterous movement. Five labels were derived to classify the dexterous movement of the child.

1. No control of any toy (NC).
2. Limited control of a toy with a single hand (LC1H). This was typically when the infant was making contact with a toy but not having gained control of it. i.e., moving the toy by hitting it, or having one hand on the toy whilst it was on the ground.
3. Full control with a single hand (FC1H), that is, when an infant was grasping the object and moving it of their own accord for a sustained period of time (approximately three seconds to differentiate from limited control).
4. Full control with two hands (FC2H), when the infant had grasped the object with both hands and manipulated it.
5. Limited control using two hands (LC2H). This final label was disregarded due to infrequent occurrence, meaning insufficient data were available for training examples.

For general body position, a further three labels where derived for classification.

1. Laying on their back, which is typically the most effective way to interact with toys when an infant is unable to sit (position back (PB)).
2. Laying on their front which allowed some relative movement; however, the infants appeared to find it difficult to interact with the toys in this position (position front (PF)).
3. In a sitting position which was typically the easiest position to interact and pick up multiple toys (position sitting (PS)).

In this context, the labels were determined based on human-understandable movements. We believe this approach is more valuable when presenting results to healthcare professionals, as it provides a clearer understanding of how the infant interacts in real-world scenarios. This method allows for a more practical and relatable interpretation of the infant’s developmental progress, which is essential for effective evaluation and intervention planning.

Data were extracted from the raw video according to the specific labels listed above. For each instance of each label, a video at the exact time point of that movement was extracted. For each time point, the video is extracted 2± s from that point, resulting in a 4 s video. The videos were rotated by 180 degrees and inverted to augment the dataset, which serves to maximize the data available for training the classifiers. This enables the classifier to learn from a more diverse set of examples, improving its ability to generalise to new, unseen data. In this case, rotating and inverting the videos simulates different viewing angles and perspectives, enhancing the classifier’s robustness and performance in classifying infant movements and postures.

**Figure 2:**
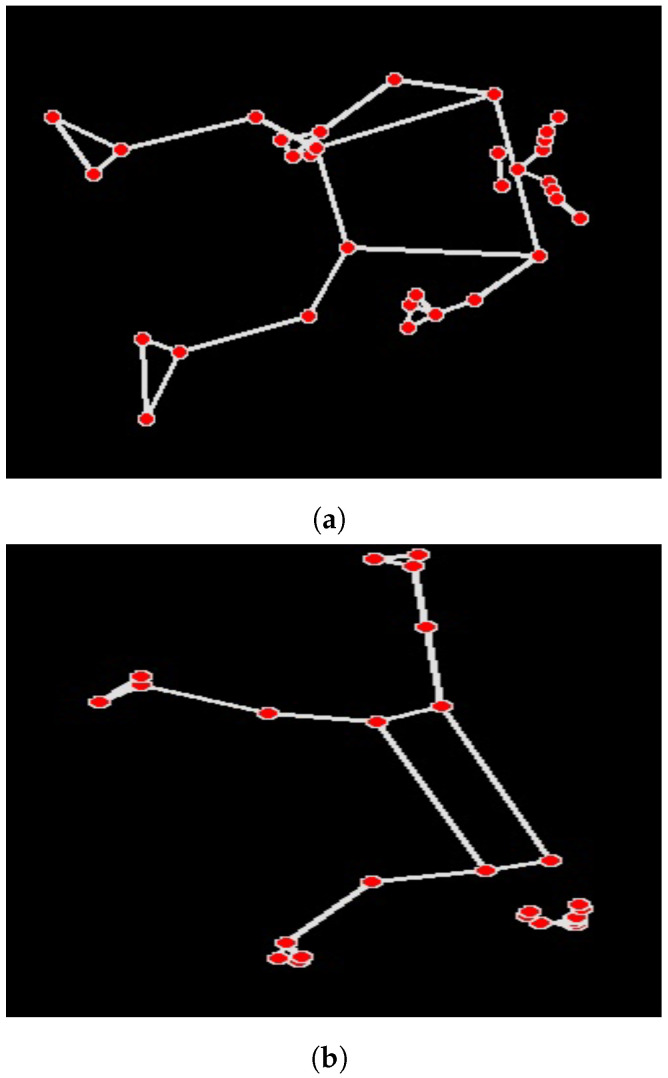
Two images of the data produced using the 2D marker-less pose estimation algorithm in media pipe. (**a**) shows all possible markers, and (**b**) shows a limited set being shown as not all points were visible to the camera.

For each of the labelled videos, the 2-D pose estimation algorithm media pipe was applied frame by frame. From this, the 33 data points (Table 1), as seen in Figure 2a, were extracted. Once this was completed for each video, the data generated by the media pipe were compiled into a moving sequence. These sequences were used to train the deep learning architectures. The data were split into two different categories (Dexterous movements (NC, LC1H, FC1H and FC2H)), and (general positioning (PS, PF, PB)). The first set of categories relate to the dexterous movements and second set related to the general body positioning.

The first experiment contained data from category one. As it is not possible to ask infants to complete specific tasks, and the purpose of this work was to classify organic movement, the class balance of the data was not equal across the four classification labels. Addressing class imbalance in classification tasks is of critical importance to ensure the accuracy and reliability of machine learning models. When the distribution of classes in a dataset is unequal, the learning algorithm may become biased towards the majority class, leading to a reduced predictive performance for the minority class. This can result in misclassification and diminished generalisation capabilities, particularly in real-world scenarios where minority class instances may carry significant consequences or hold substantial value. The breakdown of data by label was: NC = 2152, LC1H = 356, FC1H = 1340, FC2H = 2436. In order to ensure parity in each test, equal frequency of each label was required for training and validation and training. To achieve this, random sampling of the video clips from classes with occurrences greater than 356 was undertaken to balance the data across all classes. Due to the low number of 356 instances of LC1H, a large proportion of the data needed to be removed from the other classes. Consequently, each network was trained, validated and tested under two separate experimental conditions. One with the LC1H data included and one without. Although there were fewer classes when the LC1H class was removed, there was substantially more data available for each network facilitating greater generalisability.

For the second category, the position and posture data were used (PB, PF and PS) and there was a similar class imbalance within this category: for PB = 1460 instances, PF = 388 instances and PS = 1440 instances. To address this class imbalance, 388 instances were randomly sampled from both PB and PS. Although this resulted in a significant down-sampling of the data, this task appeared to be less complex than classifying dexterous movement; therefore, it would have a reduced impact on the results. Hence, all three classes were kept (PF, PB and PS) for classification in this experiment.

### Deep Learning

There are four types of deep learning neural network architectures that are used in this work. The first is the LSTM (Long term short term memory) networks [23,32,33] which is typically used for time series data. The second is the Bi-LSTM, which is similar to the LSTM except that it uses the time-series data in both directions, rather than unidirectionally. This has been shown to improve performance on time series forecasting and predictions [23,24,41]. The third neural network is the convolutional neural network (CNN), which is typically used in visual data [42,43]. However, they have also been used in combination with LSTMs to perform time series analysis and classification [44,45]. The final network is a combination of the BiLSTM network and convolutional neural network, the Bi-LSTM.

The neural networks listed above, for which the implementations can be seen in Table 2, are all trained in an identical way, using the same data (according to the current experiment being undertaken). The data are split into 70% training, 15% validation and 15% test data. The validation data are used every 200 iterations. If after 10 epochs the network has not improved according to the validation data, the training is stopped and the network with the highest validation score is returned. After training has stopped, the test set is used and performance is evaluated on that network. The maximum training time is 150 epochs; however, this was never reached due to the early stopping. The mini-batch size was 32 and the learning algorithm used for training was the adaptive moment estimation algorithm (ADAM).

Data were reported in confusion matrices. The end column at the far right of the matrix provides the percentages of all examples predicted to belong to each class correctly (in green/top number in each cell) and incorrectly (in red/bottom number in each cell) classified. This is often referred to as the precision or positive predictive value and false discovery rate, respectively. The bottom row of the matrix shows the percentage of all examples belonging to each class that are correctly (in green/top number in each cell) and incorrectly (in red/bottom number in each cell) classified. This is often referred to as recall or true positive rate and false negative rate, respectively. The bottom right cell (the intersection of prediction and belonging) provides the overall accuracy of the network by measuring the total number of classifications that were made correctly. From these confusion matrices, additional performance measures of overall precision, overall recall and the F1 score were calculated. The F1 score is calculated as the harmonic mean of precision and recall. The harmonic mean is a type of average that is particularly useful for rates and ratios. It is calculated by dividing the number of values in the data set by the sum of the reciprocals of the individual values. The harmonic mean is less affected by large values in the data set compared to the arithmetic mean. By considering both precision and recall, the F1 score balances the trade-off between the two measures, providing a more comprehensive evaluation of a classifier’s performance.

## 3. Results

The primary aim of this work was to assess feasibility of in-home monitoring of children’s neurological development through 2D video recordings, 2D pose estimation and the use of deep learning architectures. The secondary aims of this work were to compare several deep learning architectures to identify the optimal architecture for classification purposes. In order to achieve these aims, four different deep learning architectures were trained, validated and tested. The data presented here are split into two different categories (dexterous and position) with separate experimental conditions for the dexterous movement; four class and three class experiments (with and without the LC1H data, respectively).

### 3.1. Objective Performance-Dexterous Movements

**Figure 3:**
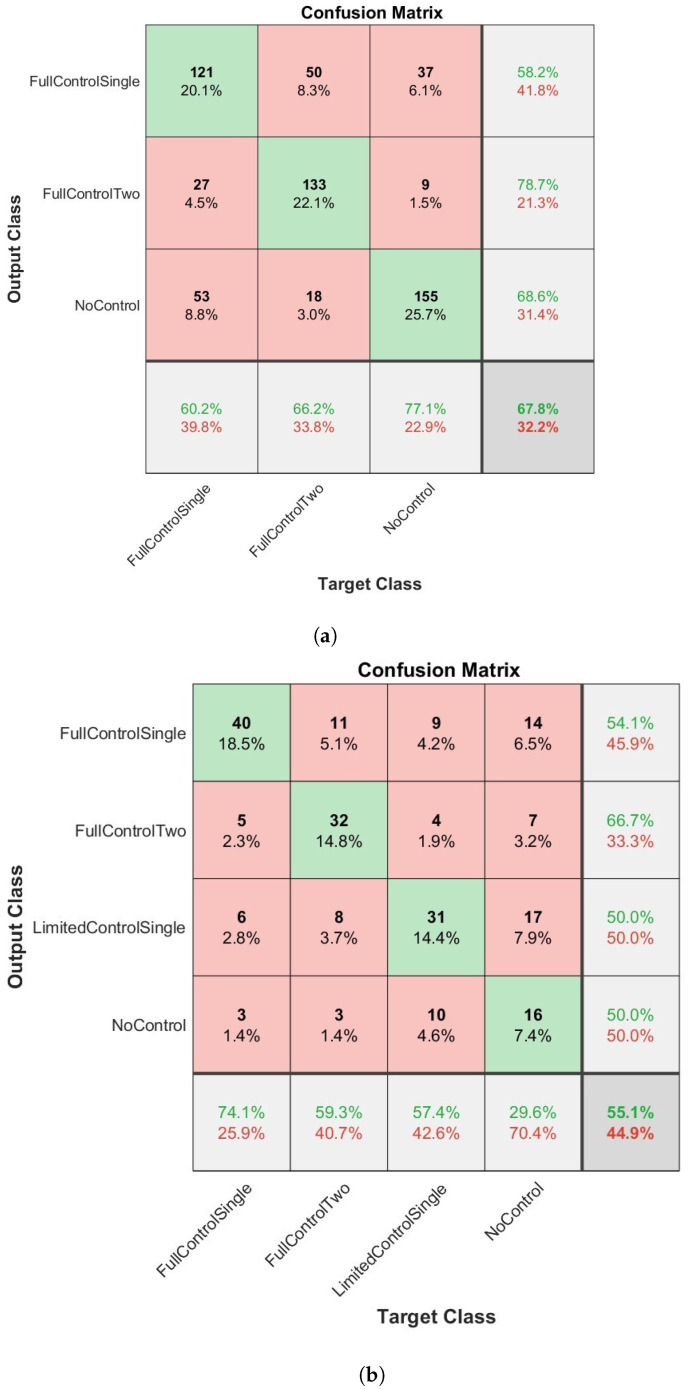
The results of the BiLSTM network when applied to the classification of infants dexterous movement. (**a**) The results from the 3 label experiment. (**b**) The results from the 4 label experiment. In bold are the highest values per column.

**Figure 4:**
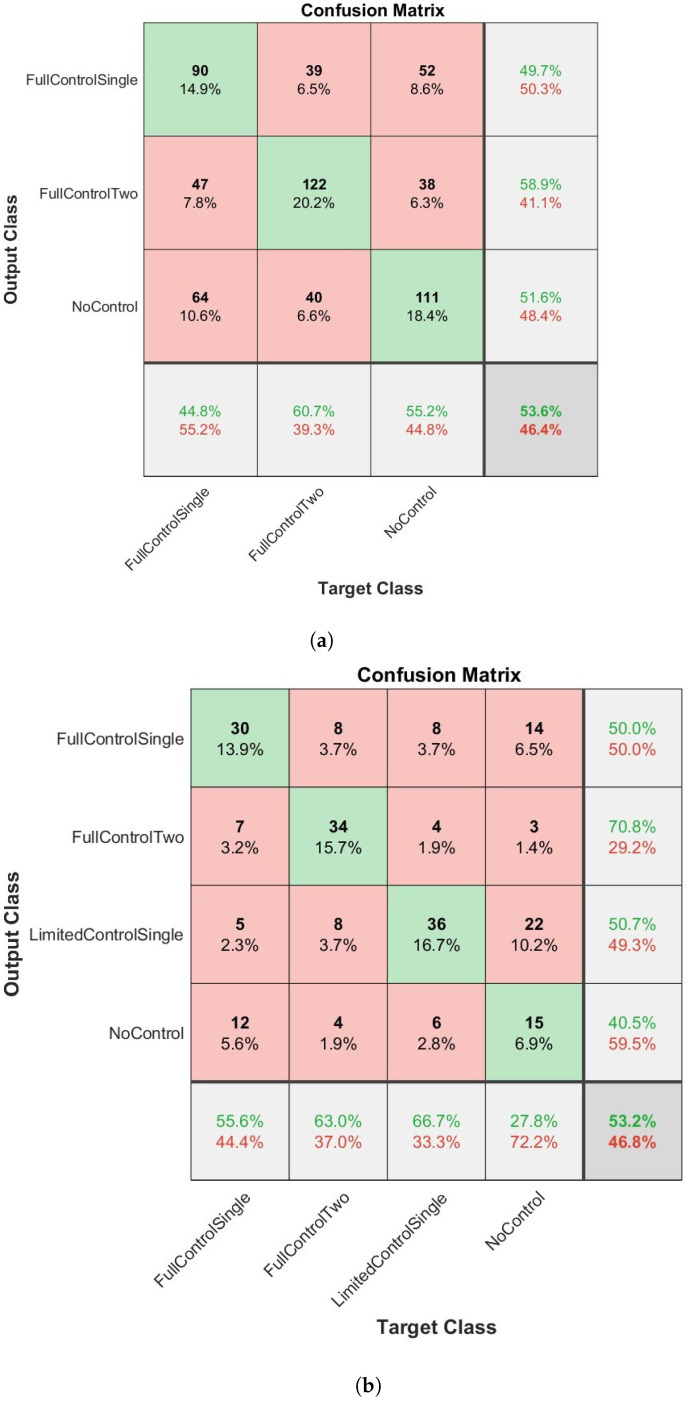
The results of the LSTM network when applied to the classification of infants dexterous movement. (**a**) The results from the 3 label experiment. (**b**) The results from the 4 label experiment.

**Figure 5:**
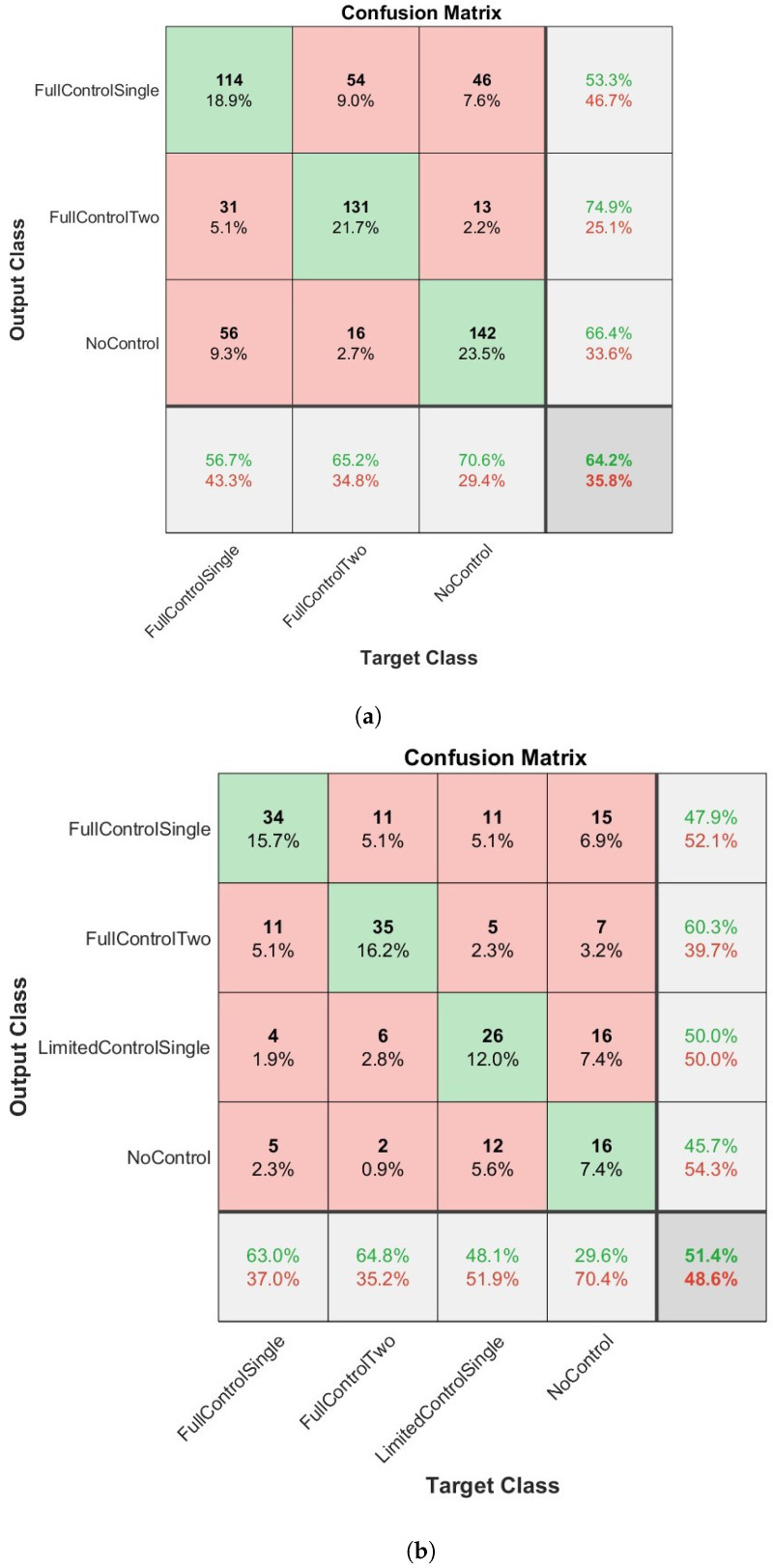
The results of the Bi-LSTMCNN network when applied to the classification of infants dexterous movement. (**a**) The results from the 3 label experiment. (**b**) The results from the 4 label experiment.

**Figure 6:**
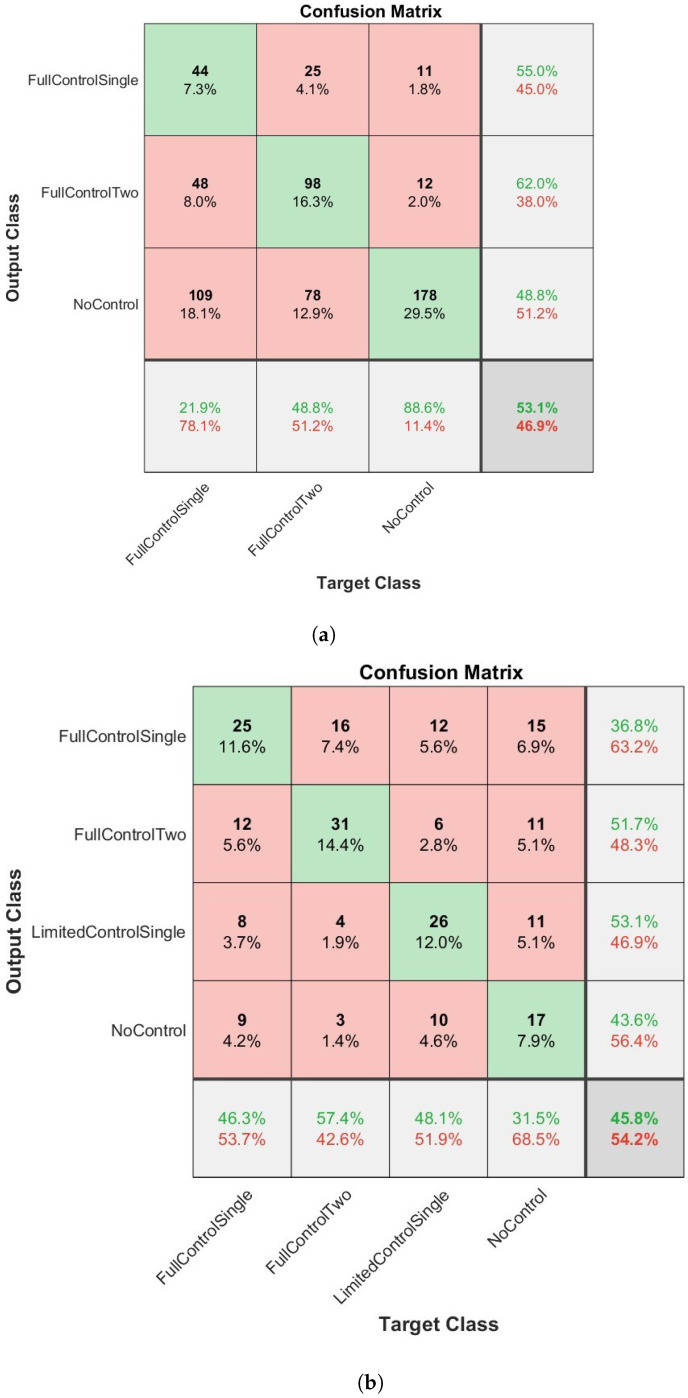
The results of the convolutional neural network (CNN) when applied to the classification of infants dexterous movement. (**a**) The results from the 3 label experiment. (**b**) The results from the 4 label experiment.

The overall results of how the four network types classified the dexterous movements of children can be seen in Figure 3, Figure 4, Figure 5 and Figure 6 and Table 3 and Table 4. Figure 3, Figure 4, Figure 5 and Figure 6 present confusion matrices for both the three class (a) and four class experiments (b). In the three class experiments, the CNN and the LSTM networks appear to have performed similarly with almost identical accuracy, precision, recall and F1 scores. However, upon closer inspection of the confusion matrices (Figure 4a and Figure 6a) the LSTM shows a greater capacity to correctly classify more evenly across the classes than the CNN. The CNN correctly classified the NC condition 88.6% of the time but only classified the FC1H class correctly 21.9% of the time and the FC2H class correctly 48.8% of the time. Essentially, the CNN’s capacity to correctly classify the NC condition so well masked the poorer performance in the other two classes, whereas the LSTM was able to classify all three classes with reasonable consistency. The Bi-LSTM-CNN performed well in the three-class experiment with similar accuracy, precision, recall and F1 scores.

In the four-class experiment, the limited availability of data for training the networks reduced the capacity of the networks to accurately classify at the same rate as in the three-class condition. Unlike in the three class condition, the Bi-LSTM-CNN did not perform to an equivalent standard of the Bi-LSTM and was outperformed by the standard LSTM with an F1 score of 50.7 compared to 53.1, respectively. The reduced amount of data available to the networks also resulted in a poorer performance for the CNN as seen in Table 3 and Table 4. In both experimental conditions, the Bi-LSTM network performed the best (Table 3 and Table 4), with the highest overall accuracy, precision, recall and F1 scores in each condition. In the three-class condition (Figure 3a), an overall accuracy of 67.8% and an F1 score of 68.2 (Table 3) were evident. The correct classification was made aon aggregate for all three classes. The two largest misclassifications by the network were when NC and FC2H were both identified as FC1H (8.8% and 8.3%, respectively). The four class condition (Figure 5b and Table 4) presented an overall accuracy of 55.1% and an F1 score of 55.1. On aggregate, the correct classification was made for all of the classes, except NC, which was mis-classified 7.4% and correctly classified 7.4% of the time. The most accurate classification was for FC1H, where only 6.5% of all target data were misclassified.

The primary aim of this study was met in the category 1 experiments as the data demonstrate that deep learning networks in combination with the 2D pose estimation algorithm media pipe [19] are capable of classifying dexterous movements of infants in relation to the toys they are playing with. In light of the secondary aims of this work, various deep learning architectures were trained, validated and tested to ascertain the most appropriate network type. In both experimental conditions, the Bi-LSTM outperformed the other network types suggesting its robustness to deal with greater numbers of classifiers and less training data.

### 3.2. Objective Performance-Position

**Figure 7:**
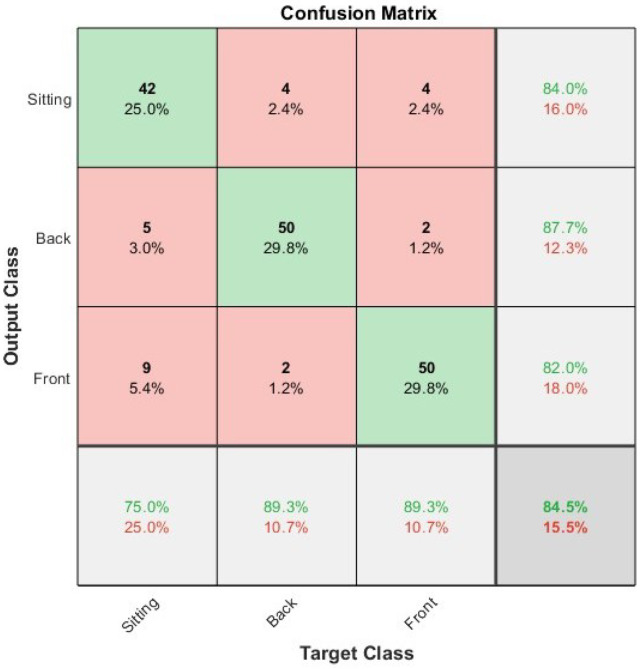
Confusion matrix presenting the results of the Bi-LSTM-CNN on classification of infant position data.

The overall performance of the four network types in relation to their capacity to classify infant positioning and posture can be seen in Table 5. This data demonstrate that all four network types were capable of accurately classifying the infant position data based on short video clips. As with the other experiments discussed in this work, the CNN was the least accurate of the networks. Figure 7 presents a confusion matrix showing the Bi-LSTM-CNN; this network demonstrated the highest level of accuracy of the four tested, achieving an overall accuracy of 84.5%. The greatest misclassification occurred between the front lying and sitting postures where nine (of the available 56) clips where incorrectly classified as front lying instead of sitting. The majority of the rest of the data were correctly classified with all categories correctly classifying data at almost 90% accuracy suggesting that infant positioning can accurately be monitored using 2D pose estimation and deep neural networks.

The overall performance of the four network types in relation to their capacity to classify infant positioning and posture can be seen in Table 5. This data demonstrate that all four network types were capable of accurately classifying the infant position data based on short video clips. As with the other experiments discussed in this work, the CNN was the least accurate of the networks.The highest accuracy was achieved by the BiLSTM-CNN, which can be seen in Table 5, achieving an overall accuracy of 84.5%. The largest misclassification is where 9 of the 56 videos of infants on their front were misclassified as sitting. For all the classes, the overall majority were classified accurately. However, in general, the classifications and accuracy were relatively uniform over the multiple classes.

### 3.3. Comparative Performance of Network Architectures

The secondary objective of this study was to evaluate the ability of four distinct architectures to classify the available data. In the dexterity experiments, the Bi-LSTM emerged as the best-performing network. In the infant position experiment, the Bi-LSTM also performed exceptionally well, with precision, accuracy, and F1 scores all exceeding 80 (Table 5). However, it was surpassed by the Bi-LSTM-CNN when analyzing positional data.

It appears that the Bi-LSTM is more suitable for classifying smaller movements and object interactions, while the Bi-LSTM-CNN excels in processing larger, more pronounced infant movements. Considering the results of all experiments conducted in this study and the minimal differences observed between the Bi-LSTM and Bi-LSTM-CNN in the positional experiments, it is recommended that the Bi-LSTM be used as the network with the highest potential for accurately classifying the widest range of data. In all experiments, the LSTM and CNN consistently underperformed in comparison to the Bi-LSTM and Bi-LSTM-CNN. Notably, there were no instances in which either the LSTM or CNN emerged as the top-performing network in any experiment, and the Bi-LSTM surpassed the performance of the LSTM in every experiment.

## 4. Discussion

The results demonstrate promising potential for this work in classifying infant movements. The top-performing networks successfully classified a substantial portion of the data across all experiments, as evidenced in Figure 3, Figure 4, Figure 5, Figure 6 and Figure 7. Notably, the overall results for the infant’s position, showcased in Figure 7 achieved an accuracy of 85%. These findings highlight the feasibility of utilizing such approaches for effectively analyzing and understanding infant movements and their developmental progress.

One of the limitations of this work which is hypothesised to have limited the overall classification rates is the camera angle and the way infants move over time. In Figure 2a, it can be seen that all 33 markers are present in the image but in Figure 2b only 20 markers are visible. This is because the infant is laying on their side and one of their arms is blocked from the camera as it is obscured by the body. A single fixed point camera was a limitation in this study due to issues with occlusion and perspective limitations. The camera’s line of sight can be obstructed, leading to missing or inaccurate data points, and the 2D representation may not fully capture the complexity of the infant’s 3D movements. This can result in limited or imprecise classification of movement patterns. Implementing multiple synchronized cameras can help overcome these limitations by reducing occlusion, providing richer spatial information, and increasing the robustness of the pose estimation and classification algorithms. By capturing a more comprehensive view of an infant’s movements and enabling 3D reconstruction, multiple cameras can enhance the accuracy of pose estimation and classification, ultimately leading to a more accurate identification of the 33 points of reference at each frame. It is predicted that this would lead to much greater classification accuracy over all models tested. Additionally, an environment which allowed infants to explore a wider range of movements, including, crawling, sitting and pulling themselves up alongside an accurate classification algorithm would be much more useful in ascertaining if infants are hitting their neurodevelopmental markers. The current work also faced challenges related to a small and potentially non-diverse sample size which could have increased the levels of class imbalance. To enhance classification rates in future work, we recommend increasing participant numbers and diversity. A larger sample size offers several benefits for the study: it would capture a wider range of movement patterns and postures, representing the natural variability in infant development, enabling better generalisation to infant populations. Additionally, a larger data set mitigates the risk of over-fitting, resulting in improved classifier performance on unseen data.

The long term and wider ranging aims for this stream of work is to facilitate the diagnosis of infant movement disorders/delays as efficiently and effectively as possible. To achieve these aims, it is vital that infants experience and demonstrate the widest range of movements available to them and that there are not overly restrictive limitations placed upon them, with respect to time, comfort, familiarity or negative impacts associated with disrupted routines. Consequently, a home-based monitoring system that allows infants to be monitored at times to suit their needs/routines in a comfortable, organic setting should facilitate the greatest results and facilitate the greatest opportunity for infants to achieve (and clinicians to observe) neurodevelopmental markers. Therefore, the development of a multi-camera system with an automated highly accurate movement classification algorithm that can be used in the home is the ultimate goal of future work associated with this project.

Additionally, if such a system is deployed in a healthcare environment, it would be of benefit to look at the transparency of the networks to better understand their decision-making process. One method of improvement would be to evaluate the formal verification of deep neural networks [46,47] to best ensure that their behaviour is in line with what we would expect.

## 5. Conclusions

The results presented in this work show the potential of 2D markerless pose estimation to classify and distinguish the movements of children both in terms of how they dexterously interact with toys and their general position. Multiple deep neural network types were evaluated, and it was found that the Bi-LSTM neural network architecture was the most performant for the dextrous experiments and the Bi-LSTM- CNN most performant for the positional experiments. This work demonstrates the potential for the autonomous, low-cost monitoring and classification of children’s movement, and for this information to be used to better inform healthcare professionals’ decision-making processes.

## Figures and Tables

**Table 1:** An example of the data points produced by media pipe.

0. Nose	12. Right shoulder	24. Right hip
1. Left eye inner	13. Left elbow	25. Left knee
2. Left eye	14. Right elbow	26. Right knee
3. Left eye outer	15. Left wrist	27. Left ankle
4. Right eye inner	16. Right wrist	28. Right ankle
5. Right eye	17. Left pinky	29. Left heel
6. Right eye outer	18. Right pinky	30. Right heel
7. Left ear	19. Left index	31. Left foot
8. Right ear	20. Right index	32. Right foot
9. Mouth left	21. Left thumb	
10. Mouth right	22. Right thumb	
11. Left shoulder	23. Left hip	

**Table 2:** The layers within the network types and topologies applied to the classification of children’s movement in this work.

Network Topology			
Bi-LSTM	Bi-LSTM-CNN	CNN	LSTM
Sequence Input Layer	Sequence Input Layer	Sequence Input Layer	Sequence Input Layer
Dropout Layer	Dropout Layer	Dropout Layer	Dropout Layer
Bi-LSTM layer (200 units)	1 × 1 Convolutional Layer	1 × 1 Convolutional Layer	LSTM layer (200 units)
Dropout Layer	Bi-LSTM layer (200 units)	Dropout Layer	Dropout Layer
ReLU Layer	Dropout Layer	ReLU Layer	ReLU Layer
Fully Connected Layer	Flatten Layer	MaxPooling layer	Fully Connected Layer
Softmax Layer	ReLU Layer	Fully Connected Layer	Softmax Layer
Output Layer	Fully Connected Layer	Softmax Layer	Output Layer
	Softmax Layer	Output Layer	
	Output Layer		

**Table 3:** The precision, recall and F1 score from the 3 label experiments. This table is a summary of the information in Figure 3, Figure 4, Figure 5 and Figure 6. In bold are the highest values per column.

	Precision 3 Class	Recall 3 Class	F1 Score
Bi-LSTM	**68.5**	**67.9**	**68.2**
LSTM	53.4	53.6	53.5
BiLSTMCNN	64.9	64.2	64.5
CNN	55.3	53.1	54.2

**Table 4:** The precision, recall and F1 score from the positional experiments from Figure 3, Figure 4, Figure 5 and Figure 6. In bold are the highest values per column.

	Precision 4 Class	Recall 4 Class	F1 Score
Bi-LSTM	**55.2**	**55.1**	**55.1**
LSTM	53.0	53.3	53.1
BiLSTMCNN	50.1	51.4	50.7
CNN	46.3	45.9	46.1

**Table 5:** The precision, recall and F1 score from the positional experiments. The corresponding confusion matrix for the biLSTM-CNN can be seen in Figure 7. In bold are the highest values per column.

	Precision	Recall	F1 Score
Bi-LSTM	83.2	82.7	82.9
LSTM	78.2	77.9	78.0
BiLSTMCNN	**84.6**	**84.5**	**84.5**
CNN	69.0	67.8	68.4
